# In vitro generation of allo-reactive human NK cells from umblillical cord blood CD34+ progenitor cells

**DOI:** 10.1186/2051-1426-3-S2-P286

**Published:** 2015-11-04

**Authors:** Yong-Oon Ahn, Ha-Ram Park, Seong-Ho Kang, Jahyang Choi, Tae Min Kim, Dae Seog Heo

**Affiliations:** 1 Cancer Research Institute, Seoul National University College of Medicine and Hospital, Seoul, Republic of Korea; 2 Cancer Research Institute, Department of Internal Medicine, Seoul National University College of Medicine and Hospital, Seoul, Republic of Korea

## 

Compare to autologous NK cells, allogeneic NK cells have more anti-tumor cytotoxicity without GVHD. Adoptive transfer of allo-reactive NK cell can be ideally used combined with other immunotherapy. However, in vivo persistency of transferred NK cells in the recipients are still an obstacle in NK cell-based immunotherapy. As most of NK cells purified from peripheral blood of donors are fully differentiated, their short lifespan might be a reason. In this study, we developed a new method of “young” human NK cell generation which can be used for immune cell therapy.

CD34^+^ hematopoietic precursor/stem cells were purified from umbilical cord blood and differentiated into NK cells in vitro. Stem cell expansion and lineage commitment can be modulated by stromal cells and exogenous cytokines. Most of CD34^+^ cells express receptors for IL-3, Flt3-L, and SCF, while a small subset expressed IL-7Rα. Without feeder cells, CD34^+^ cells were initially expanded with exogenous cytokines (IL-3, Flt3-L, IL-15, SCF, and IL-7) but most of cells were differentiated into myeloid cells. When the precursor cells were co-cultured with feeder cells (murine embryonic liver stromal cell line EL08-1D2), CD56^+^ NK cells were successfully generated after 3 weeks (x20,000 fold expansion). Interestingly, as use of feeder cells was necessary for NK cell differentiation during early days (D0 to 5), feeder cell interaction may have a role on the proliferation of early lymphoid precursors or myeloid/lymphoid fate decision of hematopoietic stem cells. When we used EL08-1D2 feeder cells transduced with human Notch ligand DLL4, most of hematopoietic precursors were differentiated into lymphoid lineage cells.

Some of generated CD56^+^ cells were non-cytotoxic group3 innate lymphoid cells (ILC3s), and ILC3s do not express most of NK cytotoxic receptors (NKG2D, NKp30, NKp46, 2B4, CD94, NKG2A, DNAM-1, and CD16). Cytotoxic NK cells can be discriminated from ILC3s based on CD11a/CD18 (LFA-1) expression, and this was confirmed using CD107a assay and IFN-γ staining. While ILC3s require SCF and IL-7 for their generation, NK cells can be successfully generated with IL-15 and Flt-3L only. As small populations of the generated NK cells express KIRs which are essential for allogeneic cytotoxicity, we epigenetically induced KIR expression using a demethylating agent 5-aza-2′-deoxycytidine during NK cell differentiation. When 5-aza-2′-deoxycytidine was treated on immature NK cells (D14 to 21), it efficiently induced KIR expression while it did not on fully differentiated NK cells (at D21). In conclusion, this new method can be used for novel NK cell based immunotherapy.

